# Mi Sleep Coach Mobile App to Address Insomnia Symptoms Among Cancer Survivors: Single-Arm Feasibility Study

**DOI:** 10.2196/55402

**Published:** 2024-04-26

**Authors:** Noel Arring, Debra L Barton, Carolyn Lafferty, Bryana Cox, Deirdre A Conroy, Lawrence An

**Affiliations:** 1 College of Nursing University of Tennessee Knoxville, TN United States; 2 School of Nursing University of Michigan Ann Arbor, MI United States; 3 Behavioral Sleep Medicine Program Department of Psychiatry University of Michigan Ann Arbor, MI United States; 4 Division of General Medicine Department of Internal Medicine University of Michigan Ann Arbor, MI United States

**Keywords:** cognitive behavioral therapy, insomnia, mobile health, breast cancer, prostate cancer, colon cancer, cancer survivor

## Abstract

**Background:**

Rates of sleep disturbance among survivors of cancer are more than 3 times higher than the general population. Causes of sleep disturbance among survivors are many and multifaceted, including anxiety and fear related to cancer diagnosis and treatments. Cognitive behavioral therapy for insomnia (CBT-I) is considered a first-line treatment for insomnia; However, a lack of access to trained professionals and limited insurance coverage for CBT-I services has limited patient access to these effective treatments. Evidence supports digital delivery of CBT-I (dCBT-I), but there is only limited evidence to support its use among survivors of cancer. Broad adoption of smartphone technology provides a new channel to deliver dCBT-I, but no prior studies have evaluated mobile dCBT-I interventions for survivors. To address the need for accessible and efficacious CBT-I for survivors of cancer, the Mi Sleep Coach program was developed to adapt CBT-I for delivery to survivors of cancer as a self-directed mobile health app.

**Objective:**

This single-arm feasibility study assessed the adherence, attrition, usefulness, and satisfaction of the Mi Sleep Coach app for insomnia.

**Methods:**

A 7-week, single-arm study was conducted, enrolling adult survivors of breast, prostate, or colon cancer reporting sleep disturbances.

**Results:**

In total, 30 participants were enrolled, with 100% completing the study and providing data through week 7. Further, 9 out of 10 app features were found to be useful by 80% (n=24) to 93% (n=28) of the 30 participants. Furthermore, 27 (90%) participants were satisfied with the Mi Sleep Coach app and 28 (93%) would recommend the use of the Mi Sleep Coach app for those with insomnia. The Insomnia Severity Index showed a decrease from baseline (18.5, SD 4.6) to week 7 (10.4, SD 4.2) of 8.1 (*P*<.001; Cohen *d*=1.5). At baseline, 25 (83%) participants scored in the moderate (n=19; 15-21) or severe (n=6; 22-28) insomnia range. At week 7, a total of 4 (13%) patients scored in the moderate (n=4) or severe (n=0) range. The number of patients taking prescription sleep medications decreased from 7 (23%) at baseline to 1 (3%; *P*<.001) at week 7. The number of patients taking over-the-counter sleep medications decreased from 14 (47%) at baseline to 9 (30%; *P*=.03) at week 7.

**Conclusions:**

The Mi Sleep Coach app demonstrated high levels of program adherence and user satisfaction and had large effects on the severity of insomnia among survivors of cancer. The Mi Sleep Coach app is a promising intervention for cancer-related insomnia, and further clinical trials are warranted. If proven to significantly decrease insomnia in survivors of cancer in future randomized controlled clinical trials, this intervention would provide more survivors of cancer with easy access to evidence-based CBT-I treatment.

**Trial Registration:**

ClinicalTrials.gov NCT04827459; https://clinicaltrials.gov/study/NCT04827459

## Introduction

There are an estimated 18 million survivors of cancer in the United States [1], a number that is expected to increase to 22 million by 2030 [2]. As many as 20% to 75% of survivors of cancer report sleep disturbances depending on the type of cancer, which is more than 3 times higher than the general population [3-6]. Results from a recent systematic review suggest the overall prevalence of sleep disturbance in survivors of cancer is 61% [7]. Survivors of breast cancer are among the most likely to report insomnia at 60% [7]. Among survivors of prostate cancer, as many as 45% reported sleep disturbance [7]. In a study of survivors of colorectal cancer, those reporting sleep difficulty was as high as 75% (75% among patients of rectal cancer and 68% among patients of colon cancer) [6].

Problems with insomnia can persist long into the survivorship period. The causes of sleep disturbance among survivors are many and multifaceted, such as anxiety and fear related to cancer diagnosis and treatments [8,9], intrusive thoughts and faulty beliefs and attitudes about sleep [10,11], nausea and hot flashes from chemotherapy [8,9], or pain after surgery [8,9]. Symptom comorbidity creates a need for interventions that address multiple sequelae, as a lack of sleep can lead to a difficult cycle of poor sleep followed by aggravated daytime symptoms [12,13].

Cognitive behavioral therapy for insomnia (CBT-I) is considered a first-line treatment for insomnia [8,14-16]. Endorsed by the American Academy of Sleep Medicine [17], components of CBT-I include (1) stimulus control (ie, limiting the occurrence of nonsleep behaviors in the bedroom), (2) sleep restriction (ie, sleep schedule management), (3) sleep hygiene (ie, behaviors and practices that facilitate sleep), (4) cognitive restructuring (ie, addressing thoughts that interfere with sleep), and (5) relaxation (ie, use of guided imagery or meditation to reduce arousal). CBT-I is a well-established, evidence-based treatment for sleep that has shown improvement in various measures of sleep continuity without known side effects [18,19]. The findings from several systematic reviews and meta-analyses support the efficacy of CBT-I among survivors of cancer with sustained improvements in insomnia over time [16,20,21], as well as fatigue, depression, and quality of life [15,16,22,23].

A lack of access to trained professionals and limited insurance coverage for CBT-I services has limited patient access to these effective treatments [24]. In response, a growing body of research is exploring the role of internet-based or digital delivery of CBT-I (dCBT-I) and finding it effective, even comparable to in-person CBT-I at improving sleep [25-27] and comorbid symptoms such as fatigue, depression, and anxiety [25]. Further, 2 studies have examined internet-based dCBT-I for survivors of cancer using the Sleep Healthy Using the Internet program. In the Sleep Healthy Using the Internet study, 28 survivors were recruited and the participants in the intervention group showed significant improvements compared to controls for multiple sleep outcomes and general fatigue [28]. The second study was larger, included a national sample of 255 Danish survivors of breast cancer, and resulted in significant improvements in multiple sleep outcomes and reductions in fatigue [29]. To date, only these 2 studies have examined internet-based dCBT-I for survivors of cancer. While the results of these studies are encouraging, one of these studies was small (total n=28) and the other study enrolled only survivors of breast cancer [28,29].

The growth of mobile smartphone technology creates opportunities to deliver dCBT-I interventions [30]. In the past decade, we have seen rapid adoption of smartphones across diverse populations with fewer disparities compared to other technologies (eg, desktop or laptop computers, or broadband internet) [31-33]. Mobile phone apps can deliver highly usable interventions without the barrier of cost [34] and provide a realistic option to provide population access to CBT-I treatment [35]. They can also help overcome barriers such as cost [34], potentially reduce disparities in access to medical care, communication barriers with medical providers, and help with symptom monitoring and management [36]. There has in fact been a proliferation of commercial smartphone apps that address insomnia. Unfortunately, in a recent review, only 1 (CBTI Coach) of the 9 commercially available apps reviewed was found to be highly adherent to evidence-based CBT-I elements and had undergone rigorous evaluation for efficacy [34]. A small number of studies have demonstrated positive effects of mobile delivery of dCBT-I, but none of these studies involved survivors of cancer [35,37-41].

To address the critical need for accessible and efficacious CBT-I for survivors of cancer, the Mi Sleep Coach app was tested in a feasibility study that assessed adherence, attrition, usefulness, satisfaction, and effect size as an intervention for insomnia. The Mi Sleep Coach app provides access to all elements of the evidence-based CBT-I strategies. To support adoption and adherence to these strategies, the app engages users in a computerized conversational dialogue with a digital agent that is modeled on the interaction between clients and trained human CBT-I experts.

## Methods

### Study Design

This single-arm feasibility study aimed to assess adherence, usefulness, satisfaction, and effect size for the Mi Sleep Coach app as an intervention for insomnia.

### Participants

Adult survivors of breast, prostate, and colon cancer who had completed curative treatment at for least 3 months and not more than 5 years before study entry and reported sleep disturbances for at least 3 months (see Textbox 1 for full inclusion or exclusion criteria) were recruited from the University of Michigan Rogel Cancer Center and through social media between March 2021 and June 2022.

**Inclusion and exclusion criteria.**

**Inclusion**

- Aged 18 years or older
- Completed curative-intent treatment (chemotherapy, surgery, or radiation) at least 3 months and not more than 5 years before study entry
- Reported trouble falling asleep or staying asleep at least 3 nights per week (most weeks) for the last 3 months
- Owned an Android phone version 8 or higher or an iPhone running iOS 11 or higher
- Able to access the internet via smartphone
- Able to read and write English
- Able to provide informed written consent

**Exclusion**

- Diagnosed with a sleep disorder other than insomnia (eg, sleep apnea, restless legs syndrome, or narcolepsy)
- Diagnosed with insomnia before cancer diagnosis
- Reported physical symptoms that interfered with sleep, such as shortness of breath, pain, hot flashes, or frequent urination
- Reported 3 or more of the following symptoms suggestive of sleep apnea: excessive daytime sleepiness; loud snoring; awakening with a dry mouth, sore throat, or morning headache; observed episodes of stopped breathing during the night; abrupt awakenings accompanied by gasping or choking
- Major psychiatric or medical condition other than cancer suspected to contribute to sleep disturbance
- Evidence of active cancer
- Currently or previously received cognitive behavioral therapy for insomnia
- Night shift workers or subject to other external restrictions on their opportunity to sleep at night

### Intervention

#### Overview

The Mi Sleep Coach app was developed by the Center for Health Communications Research (CHCR), which is based at the University of Michigan. CHCR approached the development of the Mi Sleep Coach app guided by the principles of user-centered design [42] and included iterative engagement with members of the University of Michigan Rogel Cancer Center’s patient and family advisory council for feedback on program design and message content. The Mi Sleep Coach app adapted an evidence-based clinical CBT-I program for delivery as a self-directed mobile health app for survivors of cancer. The content for the Mi Sleep Coach app is based on core principles of CBT-I, which has empirical evidence developed in multiple clinical trials [43-45]. In adapting this clinical intervention, our sleep consultant (DAC), who is board certified in behavioral sleep medicine, imagined and described what else they might do or say to the patient if they were actually with the patient throughout the day as opposed to only seeing the patient during a weekly clinic visit. The Mi Sleep Coach program was developed for cross-platform deployment on both iOS and Android operating systems. Please see Multimedia Appendix 1. The intervention topic schedule and strategies are presented in Textbox 2.

To address issues of health and technology literacy, plain language guidelines [46] were applied to inform the design of several aspects of the Mi Sleep Coach app. Examples of these plain language principles and design features included serve your audience, address the user, keep it conversational, and organize information.

**Mi Sleep Coach app intervention schedule.**

**Week 1**

- Sleep hygiene education

**Week 2**

- Personalized sleep plan
- Daily activity plan to promote sleep

**Week 3**

- Motivational interviewing (MI) for sleep plan adherence
- Updated sleep and daily activity plan

**Week 4**

- Relaxation techniques
- Updated sleep and daily activity plan with MI

**Week 5**

- Dysfunctional beliefs about sleep
- Updated sleep and daily activity plan with MI and relaxation techniques

**Week 6**

- Maintaining good sleep
- Updated sleep and activity plan with MI and relaxation
- Addressing dysfunctional beliefs

**Week 7**

- Program review

#### Serve Your Audience

The Mi Sleep Coach app provided users with a clear value proposition at initial program engagement (eg, “Think of this program as having personal Sleep Coach in your pocket to help retrain your body and mind to have better sleep.”). As part of this value proposition, the Mi Sleep Coach app explained how cancer and cancer treatments can disturb sleep, provided testimonials from survivors of cancer who had improved their sleep using CBT-I, and emphasized specific CBT-I strategies likely to address common causes of insomnia among survivors (eg, constructive worry to address anxiety about recurrence). The program tone consistently expressed empathy and encouraged patience and kindness to oneself when addressing sleep challenges.

#### Address the User

Rather than providing large volumes of information and asking users to find the specific pieces that are most personally relevant, the app directly assessed and addressed the individual’s specific needs through the application of personally tailored health messaging [47]. Examples of tailored communications (described in more detail below) include the creation of a personalized sleep and daily activity plan for each user, the application of motivational interviewing (MI) techniques to support sleep plan implementation, and the incorporation of users’ priorities and preferences in behavioral activation and cognitive reframing of dysfunctional beliefs about sleep.

#### Keep It Conversational

Prior work suggests that computer systems that engage in direct conversation-like interactions (ie, conversational or dialogue agents) can help address the needs of users with lower health literacy [48,49]. Informed by this work, the Mi Sleep Coach app was structured as a series of dialogues between the user and the program. This was accomplished by using a hybrid approach that combines sleep expert video messages with a Sleep Coach avatar (loosely modeled on the appearance of our sleep expert) that both addressed users directly. The Sleep Coach avatar functioned as a computerized dialogue agent and was able to have multiple turns of “conversation” with the user based on the user’s selection of specific fixed-choice response options. The several visual representations of the Sleep Coach avatar (ie, different facial expressions) enabled the character to exhibit different emotional states (eg, concern or caring, neutral thoughtfulness, or happiness or satisfaction) as part of interaction with users. The dialogue agent approach helped ensure that the presentation of program content was concise and easy to follow.

#### Organize Information

A series of lesson cards was integrated into the flow of conversation between the program and users. These lesson cards allowed the app to structure the presentation of information (eg, main topic and section headings) and incorporate appropriate graphics and illustrations. Narrative analogies were developed to simplify complex concepts (eg, growing a daily “sleep garden” to explain aspects of sleep biology related to circadian rhythms and sleep drive).

### App Tailoring

#### Overview

Tailored health messages in the Mi Sleep Coach app included personalized sleep plans, MI, addressing dysfunctional beliefs about sleep, and just-in-time behavioral activation.

#### Personalized Sleep Plan

The Mi Sleep Coach app asked users to complete a sleep log (items drawn from the Consensus Sleep Diary) [50] daily as well as items from the validated Sleep Needs Questionnaire (eg, Degree to which the individual is feeling tired or fatigued? Sleepy or drowsy? Taking naps or falling asleep during the day? Feeling that they are getting adequate sleep?) weekly [51,52]. The Mi Sleep Coach app then reviewed with users key metrics of their sleep from these assessments. This review focused on the user’s overall sleep efficiency (ie, time asleep or average time in bed) but also allowed the users to drill down to component and related metrics including average time asleep, average time in bed, average bedtime and consistency of bedtimes, average wake time and consistency of wake times, and average nighttime awakenings and time awake at night. At the start of each intervention week, this information was used to create a personalized sleep plan for each user. To create a personalized plan, users were prompted to pick a target wake time for the upcoming week. Based on sleep efficiency, average time in bed, and perceived sleep needs from the prior week, the Mi Sleep Coach app calculated and presented the user with a target bedtime and time in bed for the user for the upcoming week. The Mi Sleep Coach algorithm increased or decreased recommended time in bed (in 15-min intervals) to achieve a sleep efficiency (time asleep/total time in bed × 100) of 85% or more (80% for individuals aged 65 years or older) and a low reported unmet sleep need. Tailored messaging in the presentation of the sleep plan highlighted the importance of establishing a consistent bedtime and wake time and the recognition of sleep efficiency, rather than total time in bed, as a marker of healthy restful sleep. Throughout the program, users were able to review trends in program engagement (eg, number of consecutive days completing the sleep log) and progress through the program (eg, trends in sleep efficiency).

#### About MI

The Mi Sleep Coach app applied the principles of MI to support users in the implementation and adherence to their personal sleep plan [53]. Each week the program assessed the user’s motivation and confidence to adhere to the newly presented personalized sleep plan. The program reflected the user’s initial responses and then provided tailored encouragement matching the user’s level of motivation and confidence. MI Mi Sleep Coach–based activities included a review and selection (or creation) of personally relevant coping statements designed to build the user’s confidence in their natural ability to sleep (eg, Sleep is a natural process. Everybody, including me, has the natural ability to sleep. Taking control of my daily routines can improve my sleep.). The user’s preferred statements were then used as motivation- and confidence-building reminders throughout the remainder of the program.

#### Dysfunctional Beliefs About Sleep

The Mi Sleep Coach app asked users to complete the brief version of the validated Dysfunctional Beliefs and Attitudes About Sleep (DBAS) assessment [54]. User responses were used to identify that individual’s most prominent dysfunctional beliefs (eg, unrealistic expectations about sleep, catastrophizing after a night of poor sleep, misattribution of the causes of poor sleep, generalization of sleep difficulties to other areas of life). For each user’s top 3 most prominent dysfunctional beliefs, cognitive reframing was supported by providing users with a list of alternative thoughts addressing this belief. Users reviewed and selected specific alternate thoughts that were most personally relevant to them (or created their own alternative thoughts). These alternative thoughts were included as positive reminders to the patient throughout the remainder of the program. Dysfunctional beliefs were presented as “thought traps” and with the idea that specific alternative thoughts could be used to help “get out” of these traps. For example, an individual user who reported prominent unrealistic expectations regarding sleep needs (eg, I must have 8 hours of uninterrupted sleep every night in order to function) was asked to select from a list of alternative thoughts (eg, There is no one right amount of sleep that is right for everyone. In general, my body gets the sleep it needs to keep going. I sleep better when I am more relaxed about how much I sleep) or create their own personally relevant alternative. These alternative thoughts were then delivered back to them as positive reminders throughout the remainder of the program.

#### Just-in-Time Behavioral Activation

The Mi Sleep Coach app also took advantage of the just-in-time capabilities of mobile health interventions to support behavioral activation for improved sleep [55]. In addition to the personalized sleep plan (eg, target bedtime and wake time) described above, the Mi Sleep Coach app presented the users with a set of behavioral strategies that could be used at key portions of the day (eg, waking up and getting up, building sleep drive throughout the day, staying up to the target bedtime, winding down for bed, or awakening in the night). Users selected specific strategies that they would like to try at each of these times of day. The Mi Sleep Coach app then assessed experiences throughout the day and confidence to adhere to sleep-promoting practices throughout the day (eg, How easy or hard was it to get up this morning? How confident are you that you will stay up to your target bedtime?) and then delivered a push notification to users during specified portions of the day (eg, following their wake time, late morning, early afternoon, or 3 hours and 1 hour before their target bedtime) reminding them of their preferred sleep-building behavioral strategies. Users were encouraged to stop using screens and digital devices and employ relaxation strategies 1 hour before their target bedtime each day.

### Measures

An adherence rate of at least 75% was considered acceptable. Participants who engaged with the app at least 4 days per week over the 7 weeks were considered adherent.

Attrition rates were assessed weekly and for the study overall. Anyone who did not complete data collection for the full 7 weeks of the study was considered to be prematurely withdrawn and included in our attrition count. An attrition rate of 25% or less was considered feasible.

User experience and satisfaction were assessed with a 40-item investigator-developed user experience survey based upon the Unified Theory of Acceptance and Use of Technology that assessed the perceived ease of use and usefulness of the Mi Sleep Coach app features [56]. *Ease of use* is assessed by asking participants to rate 10 statements on a scale of 1 (very hard) to 4 (very easy). Participants were asked to rate how *useful* they found 10 different features (chatting with the avatar, viewing video messages, daily sleep log, personal sleep plan, reading lessons, reading stories from other people with sleep problems, creating a personal list of daily activities, creating a list of coping strategies, constructive worry activities, or deep breathing activities) of the Mi Sleep Coach app on a scale of 1 (not at all useful) to 4 (very useful). Satisfaction was assessed with statements such as “overall, I am satisfied with the Mi Sleep Coach app” and “I would recommend the Mi Sleep Coach app to other people who have trouble sleeping” using a 1 (strongly disagree) to 5 (strongly agree) scale. They were also asked to answer open-ended statements such as “please tell us what you liked about the Mi Sleep Coach app.” This was collected at baseline and week 7 via QualtricsXM [Qualtrics]), a survey software tool. We considered the app acceptable if (1) at least 75% (23/30) of participants considered at least 1 app feature at least a little useful (2 or greater on a scale of 1-4); or (2) at least 80% (24/30) of participants were satisfied (4 or greater, on a scale of 1-5) with the intervention and that at least 80% (24/30) of participants would recommend it to others (4 or greater) on a scale of 1 to 5.

The Insomnia Severity Index (ISI) is a 7-item questionnaire that measures a patient’s perceptions of the nature, impact, and severity of insomnia. This scale rates items on a Likert scale ranging from 0 to 4 for a maximum score of 28. ISI has good internal consistency (Cronbach α 0.90) [57]. Higher scores indicate more severe insomnia. A 6- to 8.4-point reduction is associated with moderate improvement in insomnia after treatment [50,58]. This measure was collected via QualtricsXM at baseline and week 7. We estimated that the effect size of the intervention would be at least as high as other effect sizes reported in the literature (approximately Cohen *d* 0.6 to 0.7) for nontherapist delivered CBT-I.

Sleep medication use was assessed by asking participants to report both prescription and over-the-counter (OTC) medications used for sleep within the last 2 weeks at baseline and week 7 via QualtricsXM.

The brief DBAS-16 is a 16-item measure that assesses faulty beliefs and unrealistic expectations about sleep using statements such as “I am worried that I may lose control over my ability to sleep” [54]. The measure has a good internal consistency of 0.77 [54]. Higher scores indicate more DBAS. This measure was collected via QualtricsXM at baseline and week 7 and within the app during week 5.

Participants were asked to report any new symptoms, illnesses, or diagnosed health problems to study team members. These adverse events were categorized using the Common Terminology Criteria for Adverse Events [59].

### Analysis

The data were summarized using descriptive statistics (mean, SD, median, percentage, and frequency). The demographic and clinical characteristics including ISI, DBAS-16, and adverse events were descriptively analyzed with effect size calculations done for the validated quantitative measure. The frequency of study participants taking prescription and OTC sleep medications at baseline and week 7 was compared using *χ*^2^ testing. A full missing-data analysis was conducted using IBM SPSS statistical software (version 27; IBM Corp).

### Ethical Considerations

This study was performed in line with the principles of the Declaration of Helsinki. Approval was granted by the Institutional Review Board of the University of Michigan on March 18, 2021 (HUM00194610). Informed written consent was obtained from all individual participants included in the study. The study team follows best practices for protecting patient privacy, including the encryption of participant data when at rest or in motion and that final study data sets are deidentified [60]. No protected health information was collected through the app. CHCR is a trusted IT service provider for the University of Michigan Health System and all CHCR programs undergo extensive information assurance and cybersecurity review including active penetration testing.

## Results

### Overview

We recruited 30 participants diagnosed with breast (n=24, 80%), prostate (n=3, 10%), and colon (n=3, 10%) cancer between March 2021 and June 2022. The mean age of participants was 54 (SD 9.1) years. Further, 27 (90%) identified as female and 3 (10%) identified as male. In total, participants reported that they were White (n=22, 73%), Black (n=5, 17%), Asian (n=4, 13%), American Indian or Alaska Native (n=1, 3%), and Hispanic or Latino (n=1, 3%). Some participants reported being of more than 1 race or ethnicity. Most (n=25, 83%) had graduated from college or had an advanced degree after college. Furthermore, 5 (17%) reported a high school diploma or some college.

### Adherence

Weekly adherence rates ranged from 96% to 100% (week 1: 97%, week 2: 97%, week 3: 100%, week 4: 100%, week 5: 100%, week 6: 100%, and week 7: 97%). The overall adherence rate for the study was 99%.

### Attrition

All 30 (100%) participants completed the study and provided data through week 7.

### Usefulness and Satisfaction

Every app feature, with the exception of reading stories from other people with sleep problems (9 out of 10), was found to be useful by 80% (24/30) to 93% (28/30) of participants. The highest-rated components were keeping a sleep log, obtaining a personal sleep plan, and reading lessons about sleep. Further, 27 (90%) of the 30 participants were satisfied with the Mi Sleep Coach app and 28 (93%) would recommend the use of the Mi Sleep Coach app for those with sleep issues.

### Insomnia

The mean change score on the ISI of 8.1 was a significant decrease from baseline (18.5, SD 4.6) to week 7 (10.4, SD 4.2; *P≤*.001; Cohen *d*=1.5; see Table 1). At baseline, all ISI scores ranged from 9 to 26, indicating mild sleep disturbance for 5 (17%) participants, moderate insomnia for 19 (63%), and severe insomnia for 6 (20%). At week 7, a total of 8 (27%) participants scored in the no clinical insomnia range (0-7) while 18 (60%) scored in the mild sleep disturbance range (8-14) and 4 (13%) scored in the moderate insomnia range (15-21). Further, 6 (20%) participants scored in the severe insomnia (22-28) range at baseline, and none scored in the severe insomnia range at 7 weeks (see Table 2).

**Table 1:** ISI^a^ and dysfunctional beliefs about sleep at baseline to week 7 among cancer survivors in the Mi Sleep Coach pilot study.

			Value, mean (SD)	Cohen *d*		*P* value
**ISI**				1.5		≤.001
	Baseline	18.5 (4.6)				
	Week 7	10.4 (4.2)				
**DBAS^b^**				1.3		≤.001
	Baseline	47 (10.8)				
	Week 7	36 (12.7)				

^a^ISI: Insomnia Severity Index.

^b^DBAS: Dysfunctional Beliefs and Attitudes About Sleep.

**Table 2:** ISI^a^ clinical ranges at baseline and week 7 among cancer survivors in the Mi Sleep Coach pilot study (N=30).

Clinical range	Baseline, n (%)	Week 7, n (%)
No clinical insomnia (ISI scores 0-7)	0 (0)	8 (27)
Mild sleep disturbance (ISI scores 8-14)	5 (17)	18 (60)
Moderate insomnia (ISI scores 15-21)	19 (63)	4 (13)
Severe insomnia (ISI scores 22-28)	6 (20)	0 (0)

^a^ISI: Insomnia Severity Index.

### Sleep Medication

At baseline, 7 (23%) participants were taking prescription medications for sleep. At 7 weeks, only 1 (3%; *χ*^2^ *P*<.001) participant was still taking prescription sleep medications. Likewise, at baseline, 14 (47%) participants were taking OTC medications for sleep. At 7 weeks, this number deceased, so that 9 (30%; *χ*^2^ *P*=.03) participants were still taking OTC sleep medications.

### About DBAS

The mean change score on the DBAS-16 of 11 was a significant decrease from baseline (47, SD 10.8) to week 7 (36, SD 12.7, *P*<.001, Cohen *d*=1.3; see Table 1). Beliefs and attitudes about sleep were moderately positively correlated with insomnia, as measured by the ISI, at baseline (*R*=0.574) but only mildly correlated with insomnia at 7 weeks.

### Adverse Events

In total, 4 participants reported adverse events while in this study. All adverse events were not related to the Mi Sleep Coach intervention. Further, 2 were mild (1 pain in extremity and 1 stomach pain) and 2 were moderate and required medication (2 allergic rhinitis).

## Discussion

### Principal Findings

This study evaluated the innovative Mi Sleep Coach mobile dCBT-I program tailored for survivors of cancer. The Mi Sleep Coach program was found to be highly feasible with 100% (N=30) of participants completing all 7 weeks of the study and providing data through week 7. Additionally, the overall program adherence rate for the study was 98%. In total, 90% (n=27) of the Mi Sleep Coach app participants were satisfied with the program and 93% (n=28) would recommend the use of the Sleep Coach app for those with sleep issues. The Mi Sleep Coach program also showed promise in addressing insomnia among survivors of cancer. Program users experienced a significant decrease in the mean change on the ISI of 8.1, from baseline (18.5, SD 4.6) to week 7 (10.4, SD 4.2; *P≤*.001), which is also represented by shifts from moderate-to-severe to mild-to-no clinical insomnia. Program users also reported a spontaneous decrease in the use of both OTC and prescription sleep medications.

The high rates of engagement and adherence to the Mi Sleep Coach program are notable. Some caution that digital interventions are particularly susceptible to attrition because participants can easily access resources a few times before abandoning them [55]. However, the high usefulness and satisfaction scores attributed to the Mi Sleep Coach app might indicate a lower likelihood of dropout. Recent systematic reviews and meta-analyses of randomized controlled trials of internet-delivered CBT have found attrition rates in the range of 21.6% to 24.7% [27,61]. While only a few of the studies focus on survivors of cancer, similar or lower dropout rates have been found in other CBT-I studies in survivors of cancer [28,29,62]. More research is needed to understand how to ensure the retention of participants in digital interventions and the possible role of attrition as a moderating variable [63]. For multicomponent CBT-I interventions, adherence varies widely depending on the details of the study. Similar to our findings, a randomized controlled trial of 30 survivors of breast cancer found adherence to 2 eHealth CBT-I components ranged from 99% to 100% [64]. However, in that study, participants met weekly in a group video conference environment, making it less like the self-managed approach used by Mi Sleep Coach. Adherence to the Mi Sleep Coach program compares favorably to reports from a fully internet-delivered CBT-I intervention for survivors of breast cancer (adherence ranges from 59.7% to 82.1%) [29]. The use of engagement strategies in the Mi Sleep Coach, such as the digital health agent, may have resulted in higher adherence rates.

The large treatment effects of the Mi Sleep Coach program (eg, Cohen *d*=1.5 for ISI) are encouraging. This effect is consistent with findings from 2 meta-analyses of largely person-delivered CBT-I for survivors of cancer that found large treatment effects (pooled effect sizes 0.77-0.78) [16,22]. The Mi Sleep Coach effects also compare favorably to the effects of dCBT-I in general where 2 meta-analyses found moderate pooled effects (eg, Cohen *d*=0.40, decrease in insomnia severity by 4.3 points) [26,27]. The finding of reduced use of sleep medications among Mi Sleep Coach participants may be particularly important. A recent national study found that approximately half of survivors of cancer with poor sleep reported using sleep medications [65]. This is of concern because the use of OTC or prescription sleep medications is associated with an increased risk of falls and fractures among older adults and survivors of cancer [66-68].

The limitations of this study include a small sample size, lack of a comparator, and the use of self-report measures for sleep. However, this study was a feasibility study and as such was designed to assess adherence, attrition, and usefulness. Additionally, sleep diaries have been used to measure the primary outcome in numerous sleep studies and have been validated against both functional and physiologic measures of sleep [69].

The results of this pilot study support that the Mi Sleep Coach app is a promising intervention for cancer-related insomnia. Future research should be conducted to test the Mi Sleep Coach app in a randomized controlled trial with sufficient control to determine its efficacy. If proven to significantly decrease insomnia in survivors of cancer in future randomized controlled clinical trials, this intervention would provide more survivors of cancer with easy access to evidence-based CBT-I treatment.

## Appendix

Multimedia Appendix 1 Mi Sleep Coach feasibility presentation.

## Abbreviations

CBT-I: cognitive behavioral therapy for insomnia
CHCR: Center for Health Communications Research
DBAS: Dysfunctional Beliefs and Attitudes About Sleep
dCBT-I: digital delivery of cognitive behavioral therapy for insomnia
ISI: Insomnia Severity Index
MI: motivational interviewing
OTC: over-the-counter

## Data Availability

The data sets generated and analyzed during this study are available from the corresponding author upon reasonable request.
