# Supplementary material for: Mi Sleep Coach Mobile App to Address Insomnia Symptoms Among Cancer Survivors: Single-Arm Feasibility Study
Source: JMIR Form Res. 2024 Apr 26;8:e55402. doi: 10.2196/55402 (PMC11087861; doi:10.2196/55402)
Supplement: Multimedia Appendix 1 [file formative_v8i1e55402_app1.pptx]

## Slide 1
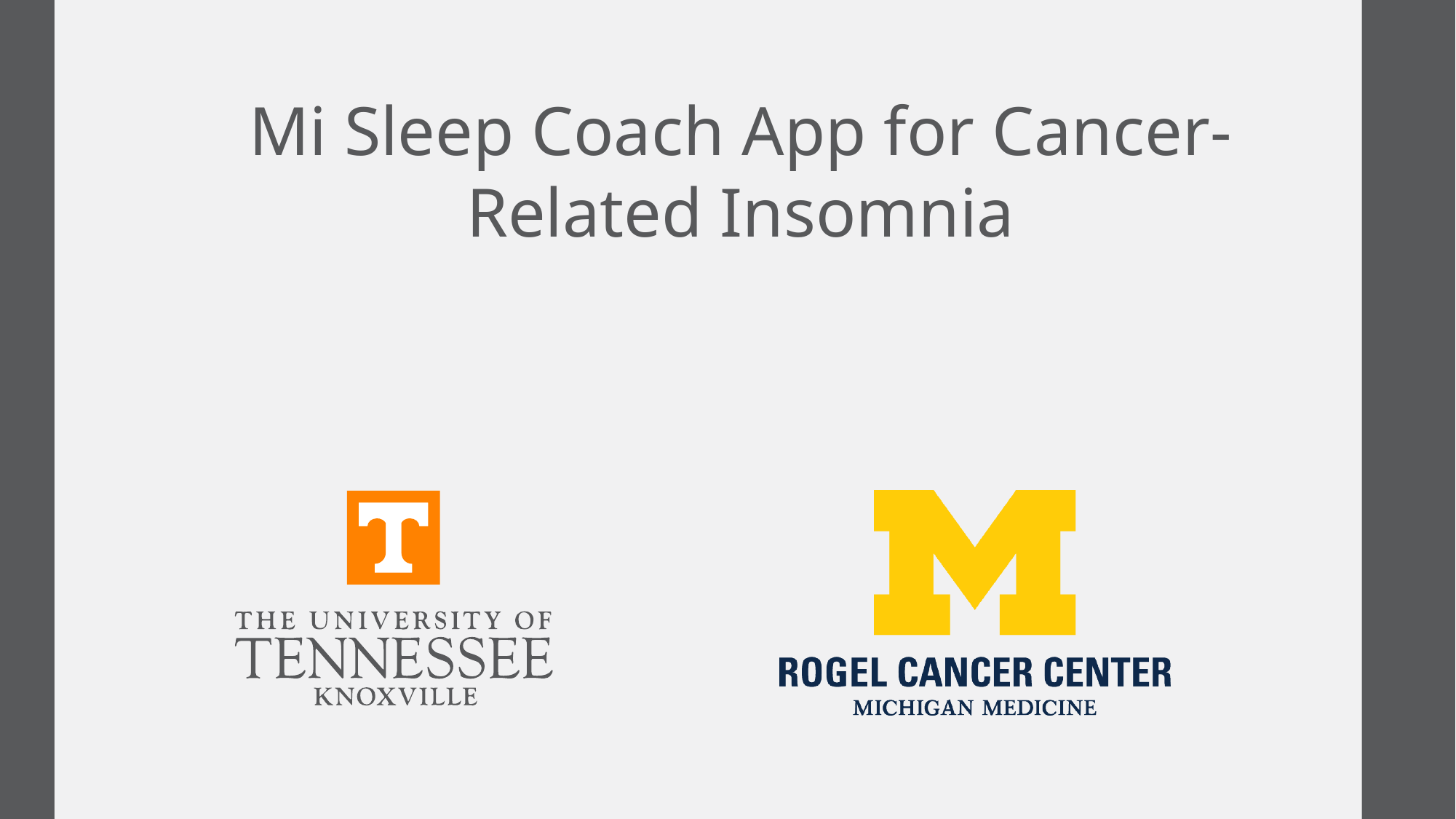

# Mi Sleep Coach App for Cancer-Related Insomnia

## Slide 2
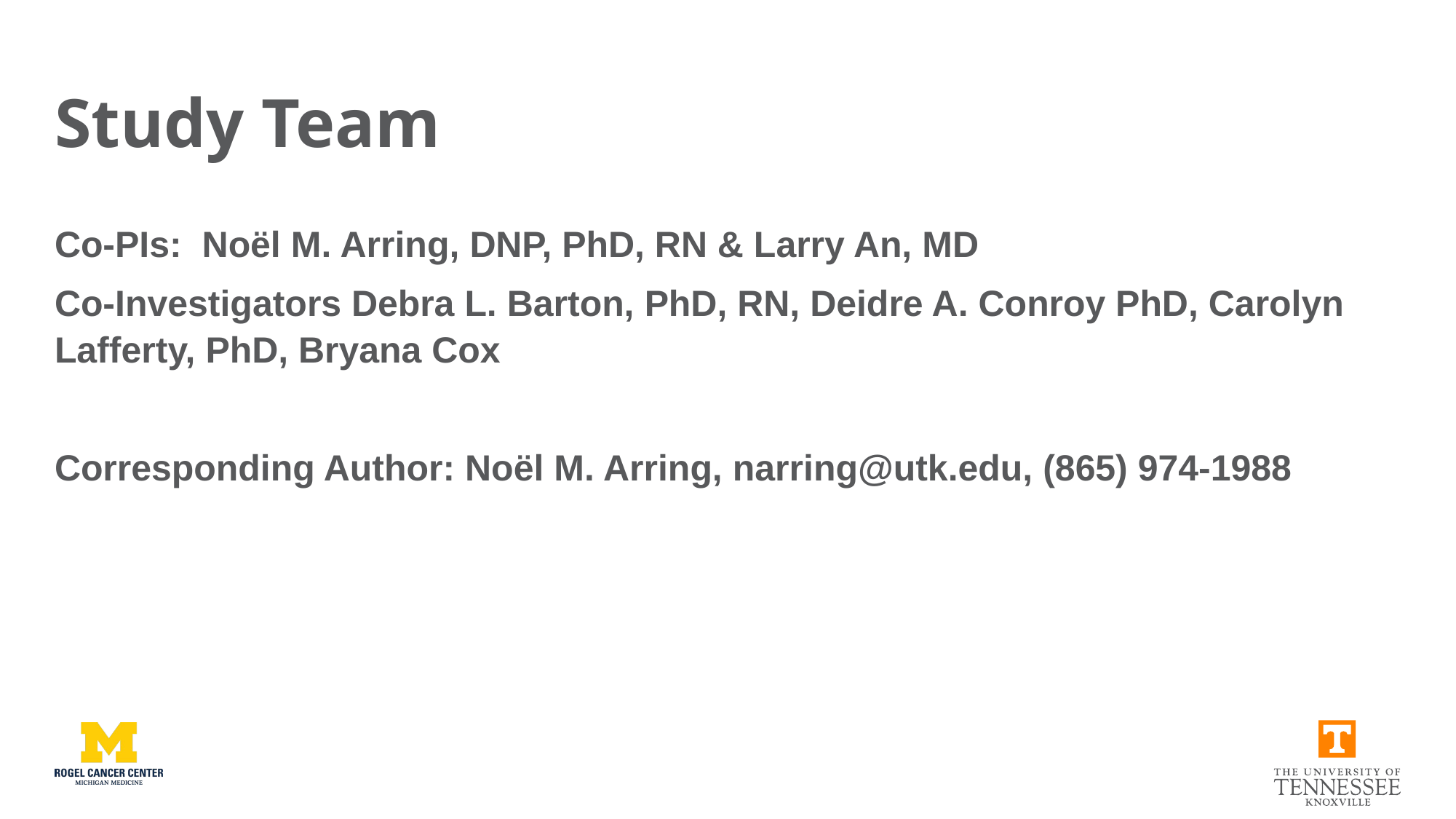

# Study Team
Co-PIs: Noël M. Arring, DNP, PhD, RN & Larry An, MD
Co-Investigators Debra L. Barton, PhD, RN, Deidre A. Conroy PhD, Carolyn Lafferty, PhD, Bryana Cox
Corresponding Author: Noël M. Arring, narring@utk.edu, (865) 974-1988

## Slide 3
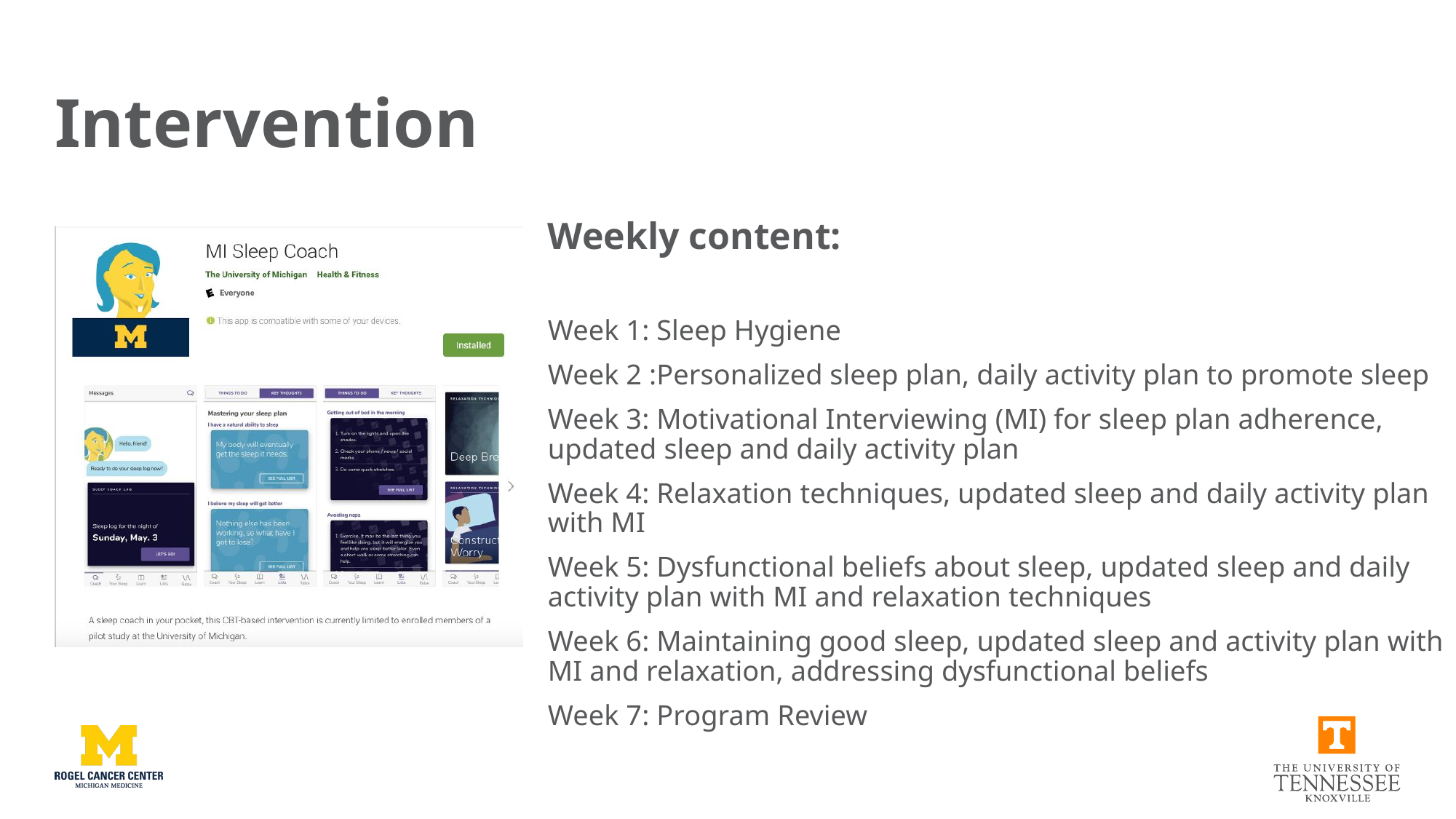

# Intervention
Weekly content:
Week 1: Sleep Hygiene
Week 2 :Personalized sleep plan, daily activity plan to promote sleep
Week 3: Motivational Interviewing (MI) for sleep plan adherence, updated sleep and daily activity plan
Week 4: Relaxation techniques, updated sleep and daily activity plan with MI
Week 5: Dysfunctional beliefs about sleep, updated sleep and daily activity plan with MI and relaxation techniques
Week 6: Maintaining good sleep, updated sleep and activity plan with MI and relaxation, addressing dysfunctional beliefs
Week 7: Program Review

## Slide 4
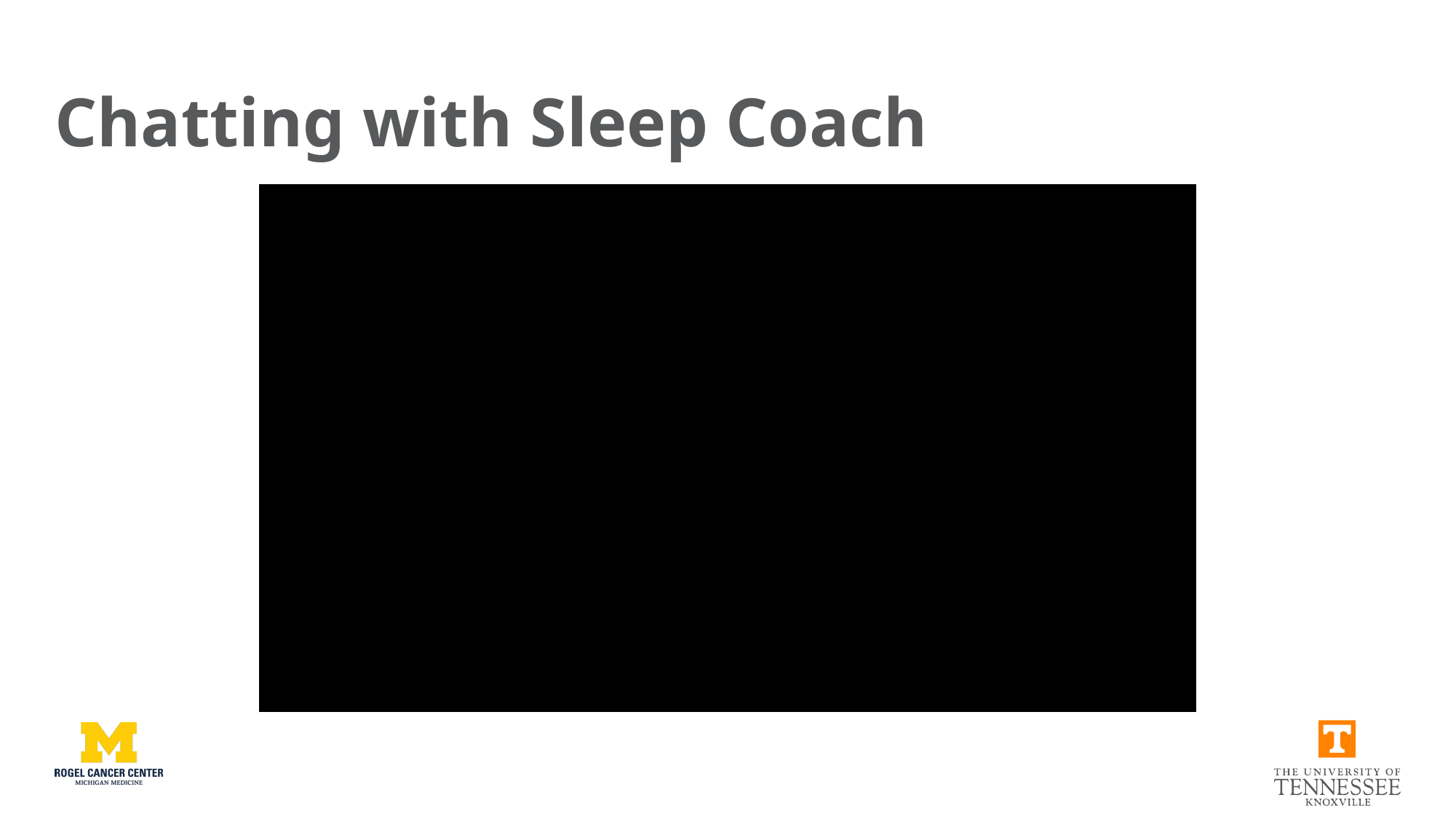

# Chatting with Sleep Coach

## Slide 5
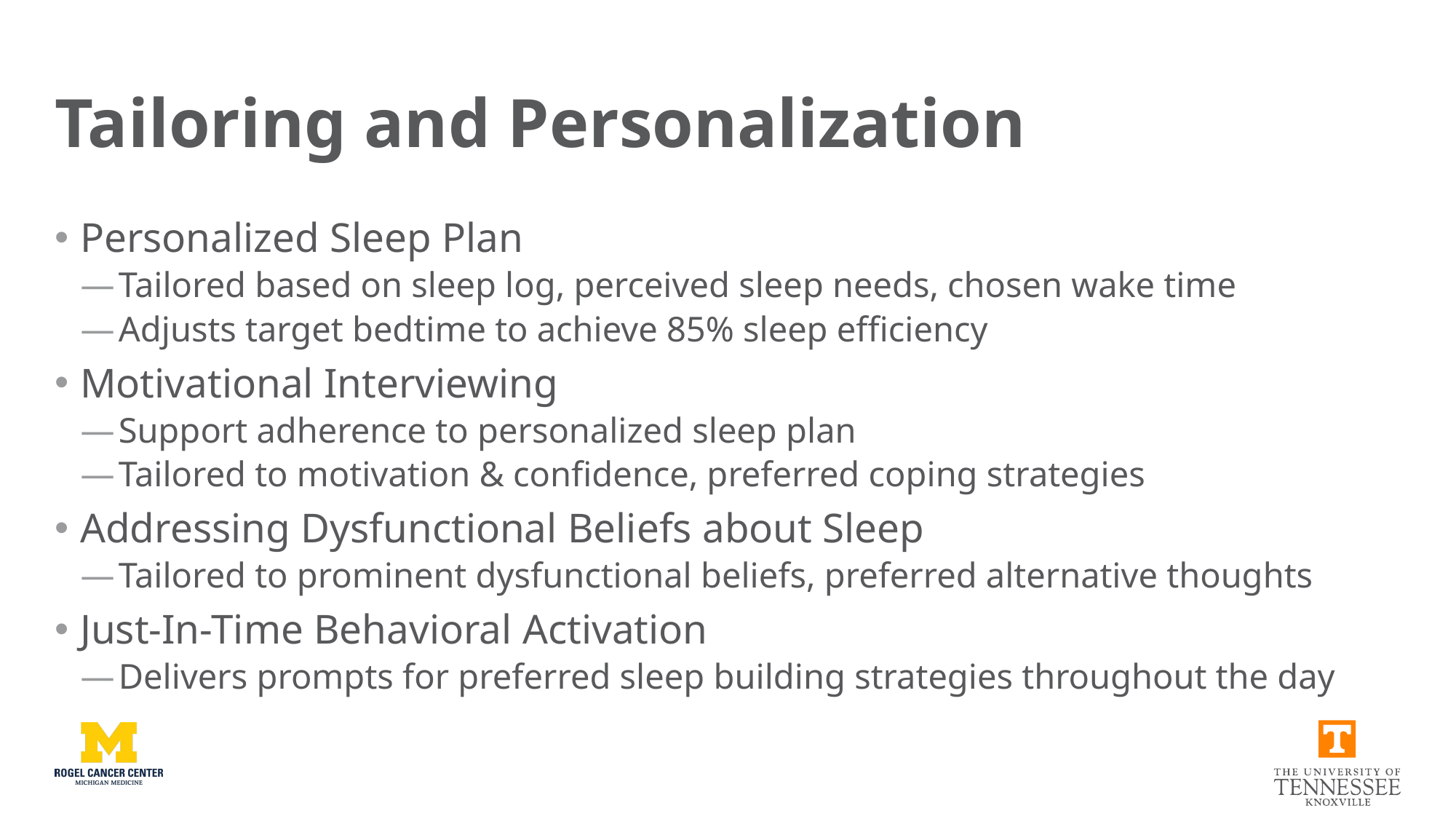

# Tailoring and Personalization
Personalized Sleep Plan
Tailored based on sleep log, perceived sleep needs, chosen wake time
Adjusts target bedtime to achieve 85% sleep efficiency
Motivational Interviewing
Support adherence to personalized sleep plan
Tailored to motivation & confidence, preferred coping strategies
Addressing Dysfunctional Beliefs about Sleep
Tailored to prominent dysfunctional beliefs, preferred alternative thoughts
Just-In-Time Behavioral Activation
Delivers prompts for preferred sleep building strategies throughout the day
